# Interleukin 6 and 8 Levels in Plasma and Fibroblast Cultures in Psoriasis

**DOI:** 10.1155/MI/2006/81767

**Published:** 2006-02-23

**Authors:** Anna Zalewska, Ewa Głowacka, Janina Wyczółkowska, Henryk Tchórzewski, Joanna Narbutt, Anna Sysa-Jędrzejowska

**Affiliations:** ^1^Department of Dermatology and Venereology, Medical University of Łódź, 5 Krzemieniecka Street, 94-017 Łódź, Poland; ^2^Department of Clinical Immunology, Polish Mother's Memorial Hospital–Research Institute, 93-338 Łódź, Poland; ^3^Centre for Medical Biology, Polish Academy of Sciences, 93-232 Łódź, Poland

## Abstract

Fibroblasts have been implicated in psoriatic inflammatory processes. The aim of the study was to evaluate soluble interleukin 2 receptor (sIL-2R), interleukin 6 (IL-6), and interleukin 8 (IL-8) plasma levels in psoriatic patients and IL-6 and IL-8 levels in fibroblast culture supernatants. Cytokines levels in plasma and supernatants were measured by ELISA. Plasma sIL-2R, IL-6, and IL-8 levels were higher before the treatment in comparison to healthy controls (*P* < 0.001) and decreased after treatment. Fibroblasts from healthy controls, psoriatic lesional skin, and noninvolved psoriatic skin, when stimulated with tumor necrosis factor alpha, released considerable amounts of IL-6 and IL-8. No significant difference between healthy controls and psoriatic fibroblasts was observed. Monitoring plasma sIL-2R levels could be employed as a reliable method of psoriasis activity. IL-8 and IL-6 plasma levels seem to reflect psoriasis activity, and treatment response, respectively. Fibroblasts are not a major source of increased IL-6 and IL-8 production in psoriasis.

## INTRODUCTION

Psoriasis is a chronic recurrent skin disease involving 1% to 2% of human population worldwide. On histology, it is characterized by hyperproliferation of keratinocytes, vascular expansion, together with leukocyte infiltration. It is widely accepted that genetic predisposition and environmental factors have a profound effect on the immune system and play a crucial role in triggering psoriatic lesion development. Psoriasis is regarded as a T_H^1^_ disorder of autoimmune background and it is postulated that changes in cytokine production both locally and systemically could be useful in monitoring activity of the disease [1–4].

Fibroblasts are the most abundant cells in the connective tissue. From the historical point of view, these cells were regarded as only quiescent elements forming stromal framework for other cells in the connective tissue [5]. Not long ago, fibroblasts were discovered to initiate the earliest molecular events, thus leading to inflammatory responses [6]. So, in view of current findings, fibroblasts should be regarded as active participants of tissue reactivity taking part in inflammatory and remodelling processes. Literature data point out fibroblast involvement in psoriatic inflammation [7, 8].

Interleukin 6 (IL-6) is a pleiotropic cytokine. Among its characteristic actions are regulation of expression of other cytokines, induction of differentiation and proliferation of normal and malignant cells, inhibition of tumor growth. IL-6 is also regarded as a major inducer of the acute-phase response [9]. IL-6 is a component of normal human skin and it was immunologically detected in basal keratinocytes, endothelial cells, many mononuclear cells, fibroblasts, and sudoriparous ducts [10]. IL-6 has been suggested to function as an autocrine mitogen in psoriatic epidermis [11]. In psoriasis, intense labelling of the cytoplasm in the vicinity of keratinocytes membranes was detected in the epidermal layers and other skin appendages. Bearing in mind that this interleukin acts synergistically with IL-1 and tumor necrosis factor alpha (TNF-α) further supports the hypothesis that IL-6 may contribute via its receptor action to epidermal growth factor (EGF) function in modulating cell hyperproliferation in psoriasis [10].

Interleukin 8 (IL-8) is the best-known chemokine. Its action is greatly enhanced by IL-1 and TNF-α. IL-8 exerts a very strong chemotactic activity towards neutrophils [12]. Gearing et al studying different cytokine levels, that is, IL-2, IL-4, IL-6, IL-8, GM-CSF (granulocyte/macrophage colony-stimulating factor) in aqueous extracts of stratum corneum from psoriatic lesions and normal heel, found out that IL-8 was the only biologically active cytokine to be elevated in psoriatic lesional extracts [13].

The aim of the study was to evaluate sIL-2R, IL-6, and IL-8 plasma levels in psoriatic patients and Il-6 and IL-8 levels in fibroblast cultures.

## MATERIALS AND METHODS

The study comprised 106 patients (30 females, 76 males), aged 19–79 years (mean 44.9 ± 13.6 years) hospitalized at the Department of Dermatology and Venereology, Medical University of Łódź, because of psoriasis vulgaris of moderate-to-severe course. The control group comprised 40 healthy volunteers (17 females, 23 males), aged 22–69 years (mean 46 ± 13.5 years). Psoriatic patients presented an active disease and the last exacerbation lasted from 2 to 8 weeks (mean 4 ± 1.6 weeks). The patients took only emollients and keratolytic drugs before admittance to hospital. As for comorbidity, hypertension was the most often discovered in psoriasis patients (52 out of 106), then diabetes type II (7 out of 106), and peptic ulcer (4 out of 106).

Blood was collected into pyrogen-free EDTA tubes in the morning of the second day after admittance to hospital and 3 weeks after inpatient treatment, centrifuged within 30 minutes of collection (at 1000 x*g*), and plasma was frozen in −70°C until further evaluated.

On the days of blood collection, clinical severity of the disease was evaluated by PASI score (range 1–72 points) [14, 15]. Before inpatient treatment, PASI ranged from 7.2 to 29.8 (mean 16.7 ± 5.7), and 3 weeks thereafter ranged from 2.8 to 19.5 (mean 9.3 ± 4.1).

In hospital, the following treatment methods were employed: Ingram method (ie, anthralin plus UVB-NB irradiation: 311 nm, total cumulative dose mean 17.3 J/cm^2^) received by 58 patients, anthralin alone received by 10 patients, Goeckerman method (ie, tar plus UVB-NB irradiation: 311 nm, total cumulative dose mean 18.5 J/cm^2^) received by 10 patients, methotrexate (total dose mean 27.5 mg administered orally) received by 28 patients.

In psoriasis vulgaris patients, a 3 mm punch biopsy was taken from a representative psoriatic lesion located on the external surface of the forearm from the plaque centre and from perilesional noninvolved skin situated about 4–5 mm visible by the naked eye psoriatic plaque edge. In the control group, biopsies were taken once from the normal skin of the forearm. The tissue was digested with 0.25% collagenase and 0.05% deoxyribonuclease I (Sigma BioSciences, St Louis, Mo, USA) in 1 mL MEM (Biomed, Lublin, Poland) supplemented by 20% foetal calf serum (FCS; Hungarpol, Warsaw, Poland), 25 mM HEPES, 2 mM L-glutamine, 100 units/mL of penicillin, and 100 μg/mL of streptomycin (complete MEM, all from Sigma BioSciences, St Louis, Mo, USA), at 37°C for 24 hours. Cells released during tissue digestion were transferred to tissue culture flasks supplemented with 5 mL of complete MEM and cultured in CO_2_ incubator. The medium was changed twice a week until confluent fibroblast cultures were obtained. Cells of passages 3 to 4 were used for the current study. Three different populations of fibroblasts were used: from normal skin of the healthy controls, from lesional skin of psoriatic patients (psoriatic skin), and from perilesional skin of psoriatic plaques (noninvolved psoriatic skin). Fibroblasts (10^5^ cells/mL) were incubated with either TNF-α (10 ng/mL) or IL-8 (0.1 μg/mL) for 6 hours, washed in culture medium and subsequently suspended in it, and further incubated in pure culture medium for 21 hours when supernatants were collected and frozen in −70°C until further evaluated.

In plasma sIL-2R, IL-6, and IL-8 and in supernatants, IL-6 and IL-8 were assessed by ELISA method (sIL-2R by Endogen kits—Pierce Biotechnology, Inc., Rockford, Ill, USA; IL-6 and IL-8 by OptEIA Sets—Pharmingen, BD Biosciences, San Jose, Calif, USA) according to the manufacturers instructions.

All the patients and individuals participating in the study gave their informed consent according to the Medical University of Łódź Bioethic Committee requirements.

## STATISTICAL ANALYSIS

The obtained results were expressed as minimal and maximal values (range) and median value (Me). Numerical variables distribution was assessed by λ-Kolmogorow test. Comparisons between groups were performed using Mann-Whitney and Pearson correlation coefficient (*r*). A *P* value less than 0.05 was considered to be statistically significant.

## RESULTS

### Plasma measurements

We observed significantly increased plasma sIL-2R and IL-6 levels before treatment than 3 weeks after inpatient treatment and also when comparing with the healthy controls (both *P* < 0.001). No significant differences in the above cytokine plasma levels were found between psoriatic patients after treatment and healthy controls (*P* > 0.05). IL-8 plasma levels before treatment were also significantly higher than after treatment (*P* < 0.01) and in comparison to healthy controls (*P* < 0.001). Still after 3 weeks of treatment in psoriatic patients, IL-8 plasma levels were significantly higher than in the healthy controls (*P* < 0.01) (Table 1).

**Figure 1:**
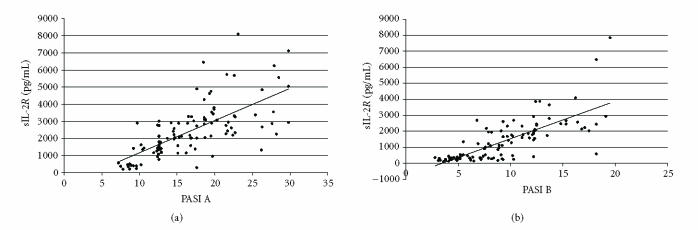
Correlation between sIL-2R plasma levels and PASI score before (PASI A) and after (PASI B) the treatment (*y* represents linear regression equation; *R^2^* represents determination coefficient): (a) PASI A, *y* = 187.77*x* − 708.51, *R^2^* = 0.4725; (b) PASI B, *y* = 234.67*x* − 814.12, *R^2^* = 0.5604.

Analysis of correlations between clinical parameters such as sex, age of the patients, and their disease development, time of the last exacerbation, PASI score, and sIL-2R, IL-6, and IL-8 was performed. Both before and after treatment, there was a positive correlation between sIL-2R and PASI score (*r* = 0.69, *P* < 0.001, and *r* = 0.75, *P* < 0.001, resp) (Figure 1). Also a positive correlation was observed between age of the patients and sIL-2R plasma levels after the treatment (*r* = 0.22, *P* < 0.01).

Next, before treatment, a positive correlation between plasma IL-8 levels and PASI score was observed (*r* = 0.25, *P* < 0.01). Additionally, also a positive correlation, however, after treatment between plasma IL-6 and PASI score was found (*r* = 0.19, *P* < 0.05). What is more, before treatment, also a positive correlation between sIL-2R and IL-8 plasma levels was discovered (*r* = 0.23, *P* < 0.05).

### Fibroblast cultures

Stimulation by either IL-8 or TNF-α caused huge production of IL-8 by all types of fibroblasts in comparison to baseline conditions (70–100 times greater) (*P* < 0.001). Also stimulation with TNF-α led to considerable increase (10–20 times) in IL-6 release by all fibroblast populations in comparison to baseline and IL-8 stimulation (*P* < 0.001) (Table 2).

At baseline, healthy controls fibroblasts released significantly higher amounts of IL-6 than noninvolved psoriatic skin fibroblasts (*P* < 0.05). When stimulated with IL-8, no statistically significant difference in IL-6 release was observed between all the studied fibroblast cultures. However, when stimulated with TNF-α, healthy controls fibroblasts released increased amounts of IL-6 in comparison to noninvolved psoriatic skin fibroblasts (*P* < 0.05).

At baseline, no statistically significant differences in IL-8 release were observed between all types of fibroblasts. However, when stimulated with IL-8 or TNF-α, noninvolved psoriatic skin fibroblasts released significantly lower amounts of IL-8 than both psoriatic skin fibroblasts and healthy controls fibroblasts (*P* < 0.05) (Table 2).

## DISCUSSION

Interaction between T cells and keratinocytes is important in pathogenesis of psoriasis by secretion of proinflammatory cytokines and growth factors in psoriatic skin. Cellular receptor for IL-2 is expressed on activated T cells and can be shed from the cells and measured as a soluble protein (sIL-2R) [16]. Several cytokines have been increased in psoriasis, either at local or systemic level or both including TNF-α, IL-2, IL-6, IL-8, interferon gamma (IFN-γ), and transforming growth factor alpha (TGF-α) which are regarded as hallmark cytokines in psoriatic cytokine network [4, 7, 17–19]. For decades, fibroblasts seemed to be only “reserved” to active participation in fibrotic processes. Nowadays however, it is postulated that these cells could actively participate in many immunological processes including inflammation. However, some reports deal with a possible involvement of fibroblasts in the development of this disease [7, 8, 20–22].

In our group of patients, plasma sIL-2R, IL-6, and IL-8 levels were significantly increased when comparing with healthy controls, and subsequently they fell down parallel with successful treatment in all the treatment groups. Our results are in line with previous reports [23–33], but some authors argue that isolated topical antipsoriatic treatment is unable to lead to systemic changes [16]. However, Naldi reported that conventional therapies despite being widely used for decades, have not been thoroughly studied [34].

So, it seemed quite reasonable to examine plasma sIL-2R, IL-6, and IL-8 levels and their correlation with different clinical parameters on quite numerous group of psoriasis vulgaris patients. In our study, plasma sIL-2R levels, both before and after inpatient treatment, correlated in a positive way with PASI. Also we demonstrated a positive correlation between age of the patients and plasma sIL-2R levels after treatment. This could result to some extent from the observation that the older the age of the patients, the longer duration of the disease and maybe similar pattern of response as regards disease activity evaluation. Thus, the older the patients, the higher plasma sIL-2R levels after successful antipsoriatic treatment. Plasma sIL-2R levels could be regarded as a marker of treatment response in psoriatic patients and employed in clinical practice.

Literature data point out increased serum IL-6 and IL-8 levels in psoriasis [11, 35–38]. Some of the reports also demonstrate that either lesional or serum levels of these cytokines reflect to some extent disease activity and treatment response [12, 39, 40]. In our group, a positive correlation between plasma sIL-2R and IL-8 levels before treatment was demonstrated suggesting that IL-8 could be regarded as an additional, apart from sIL-2R, indicator of psoriasis activity. We also observed a positive correlation between plasma IL-6 and sIL-2R levels after treatment. These results seem to point out IL-6 as an indicator of treatment response.

Debets et al, studying normal and psoriatic fibroblasts and their secretion of IL-1, IL-6, IL-8, and TNF-α, concentrated on culture conditions, and discovered that FCS, inactivated FCS, and human serum completely inhibited the expression of IL-6 mRNA in all lesional psoriatic fibroblasts [8]. The authors discovered that psoriatic and normal fibroblasts produced negligible amounts of IL-1 and TNF-α. Psoriatic fibroblasts secrete low but increased amounts of IL-6 compared to normal fibroblasts under serum-free conditions and production of IL-8 was comparable to that of IL-6. The authors discovered that TNF-α stimulation leads to considerable IL-6 release by normal fibroblasts and much less pronounced release of IL-8. Our experiments, however, show that stimulation of IL-8, which is abundantly expressed in psoriatic lesions and acts as a very strong chemoattractant towards neutrophils gathered in psoriatic lesions, caused a huge release of IL-8 by all types of fibroblast. This could suggest that fibroblasts per se are very sensitive to this cytokine and are able to further release IL-8 in an autocrine manner. Also stimulation with TNF-α led to huge response in IL-8 release by all the studied fibroblast populations.

The obtained results demonstrated that stimulation of fibroblasts with either IL-8 or TNF-α could lead to potentiation of inflammatory response. If we take a try to extrapolate the obtained results to in vivo conditions, one could assume that fibroblasts are involved in inflammatory response in the development of psoriatic plaques, however not as most powerful participants, which seems to be in agreement with the literature data [22, 41, 42].

We did not observe significant differences in cytokine release between fibroblasts obtained from healthy controls skin and fibroblasts from lesional psoriatic skin. Other authors also did not find difference in many aspects of normal and psoriatic fibroblasts [8]. But is it worth pointing out that fibroblasts from perilesional skin of psoriatic plaques demonstrated significant differences in cytokine release compared to normal fibroblasts from healthy controls, which could suggest that direct surroundings of well-developed psoriatic plaques are already affected by pathological processes.

Among shortcoming of the present study one has to admit that unfortunately due to ethical reasons, measurements of interleukin levels in cell supernatants were performed only before the treatment. As for plasma level evaluation, two measurements were performed in a 3-week interval. We could have expanded the time interval, however inpatient treatment usually does not last longer and we wanted the patients to adhere to the employed treatment as strictly as possible. Bearing in mind that about 30% to 40% of patients demonstrate nonadherence, evaluation of inpatients when they are separated from everyday worries and concentrated almost only on treatment issues is most desired and reliable [43, 44].

Worth mentioning is also one thing, that is, that extrapolation of the obtained results in vitro into in vivo conditions should be extremely cautiously performed. Direct and too brave conclusions could be difficult to draw and most confusing to compare with data obtained in different laboratory conditions. It could be argued that plasma levels of examined cytokines were already performed and published a few times but one has to bear in mind also the fact that cytokine evaluation results may vary due to different assays, individual variation in stage of the disease, demographic differences, and coexisting pathologies [29]. It should also be stressed that in context of fibroblasts, regarded as active participants of inflammatory response, the obtained results seem to be of value.

In conclusion, the obtained results confirm usefulness of plasma sIL-2R levels evaluation in monitoring psoriasis vulgaris activity during treatment. Additionally, they also support the notion that fibroblasts per se could be regarded as important elements of inflammatory reactions in human skin, however, they should rather not be treated as a major source of increased production of IL-6 and IL-8 in psoriatic skin.

## Figures and Tables

**Table 1:** Comparison of sIL-2R, IL-6, and IL-8 levels in plasma of psoriasis vulgaris patients before treatment and three weeks thereafter compared with healthy controls.

Parameter	Statistical parameters	Psoriatic patients		Healthy controls (c)	*P* level

		Before treatment	After 3 weeks of		
		(a)	treatment (b)

sIL-2R (pg/mL)	range	203–8103	104–7851	437–2101	(a)-(b) *P* < 0.001

	Me	2166	1103	897	(a)–(c) *P* < 0.001
					(b)-(c) *P* > 0.05

IL-6 (pg/mL)	range	0–305	0–74	0–7	(a)-(b) *P* < 0.001

	Me	4	1	1	(a)–(c) *P* < 0.001
					(b)-(c) *P* > 0.05

IL-8 (pg/mL)	range	0–20	0–11	0–1	(a)-(b) *P* < 0.001

	Me	2	0	0	(a)–(c) *P* < 0.001
					(b)-(c) *P* < 0.01

**Table 2:** Comparison of IL-6 and IL-8 levels in supernatants obtained from cultures of different types of fibroblasts. (a) Healthy controls: fibroblasts from normal skin of healthy volunteers; (b) psoriatic skin: fibroblasts from lesional skin of psoriatic patients; (c) noninvolved psoriatic skin: fibroblasts from perilesional skin of psoriatic patients.

Evaluated cytokine	Stimulus	Statistical parameters	Type of fibroblast culture			*P* level

			Healthy controls	Psoriatic skin	Noninvolved	
			(a)	(b)	psoriatic skin (c)

IL-6 (pg/mL)
	Control	range	560–2265	300–3350	215–1550	(a)-(b) *P* > 0.05
						(a)–(c) *P* < 0.05
		Me	1444	975	462	(b)-(c) *P* > 0.05

	IL-8 (0.1 μg/mL)	range	640–3390	465–3225	270–5465	(a)-(b)-(c) *P* > 0.05

		Me	1225	967	795	

	TNF-α (10 ng/mL)	range	3570–94000	2890–32450	850–31880	(a)-(b) *P* > 0.05
						(a)–(c) *P* < 0.05
		Me	25890	7738	4105	(b)-(c) *P* > 0.05

IL-8 (pg/mL)
	Control	range	1540–8690	435–6080	435–6624	(a)-(b)-(c) - *P* > 0.05

		Me	2924	2803	1238	

	IL-8 (0.1 μg/mL)	range	121780–405000	222684–416704	22412–2879913	(a)-(b) *P* > 0.05
						(a)–(c) *P* < 0.05
		Me	312550	344399	199391	(b)-(c) *P* < 0.05

	TNF-α (10 ng/mL)	range	126900–540872	69866–412728	25886–290981	(a)-(b) *P* > 0.05
						(a)–(c) *P* < 0.05
		Me	319359	301979	123167	(b)-(c) *P* < 0.05
